# Minimize the Percentage of Noise in Biomedical Images Using Neural Networks

**DOI:** 10.1155/2014/757146

**Published:** 2014-07-17

**Authors:** Abdul Khader Jilani Saudagar

**Affiliations:** Department of Information Systems, College of Computers and Information Sciences, Al Imam Mohammad Ibn Saud Islamic University (IMSIU), P.O. Box 5701, Riyadh 11432, Saudi Arabia

## Abstract

The overall goal of the research is to improve the quality of biomedical image for telemedicine with minimum percentages of noise in the retrieved image and to take less computation time. The novelty of this technique lies in the implementation of spectral coding for biomedical images using neural networks in order to accomplish the above objectives. This work is in continuity of an ongoing research project aimed at developing a system for efficient image compression approach for telemedicine in Saudi Arabia. We compare the efficiency of this technique against existing image compression techniques, namely, JPEG2000, in terms of compression ratio, peak signal to noise ratio (PSNR), and computation time. To our knowledge, the research is the primary in providing a comparative study with other techniques used in the compression of biomedical images. This work explores and tests biomedical images such as X-rays, computed tomography (CT), magnetic resonance imaging (MRI), and positron emission tomography (PET).

## 1. Introduction

Medical image compression (MIC) is a basic but important factor in the telemedicine where medical image samples are transferred over a channel from one location to another for remote analysis. In such cases the observer needs accurate information as near as the original sample to have correct decision. However, in the current scenario to transmit this large volume of image data, a higher resource such as high bandwidth is needed. Improving allocated bandwidth is not an economical solution; hence advanced compression approaches are to be developed so as to compress the medical samples with the lowest level of errors and in less time.

For the compression of medical images various image compression approaches were proposed in the past. The conventional image compression approaches such as JPEG [1], JPEG-2000 [2], SPHIT [3], EBCOT [4], and lifting scheme [5] were proposed earlier. These approaches are majorly categorized under lossy or lossless compression schemes. In lossy compression [6] the information is not accurately retrieved at the receiver side resulting in low PSNR. These methods are basically suitable for faster transmission approach. In various scenarios where degradation of image is not tolerable, lossless compression schemes were proposed. Lossless compression scheme [7] is a method that allows the exact accurate original data to be reconstructed from the compressed data. A scheme such as wavelet-based compression with adaptive prediction [8] is a lossless approach of image compression. This scheme is mainly used to achieve higher compression ratio. For obtaining a lossless compression in [9] a lifting scheme is suggested based on adaptive threshold.

Lossy and lossless compression schemes were found limited while being applied over medical image processing. In order to retrieve the sample with the highest accuracy and faster transmission, for this reason artificial intelligence based approaches were proposed. These artificial neural networks (ANN) have been applied to medical image compression problems, due to their superiority over traditional methods when dealing with noisy or incomplete data. Artificial neural networks (ANN) approaches are accurate in making decisions but are computationally effective. Laura et al. [10] have presented a technique in the medical application of image compression using neural networks, which allows carrying out both compression and decompression of the images with a fixed ratio of 8 : 1 and a loss of 2%. Here backpropagation network is created for correspondence functional calculations of input and output patterns.

A similar approach in [11] with backpropagation algorithm using feed-forward neural (FFN) network is suggested. In this method medical image compression is carried out by calculating coupling weights and activation values of each neuron in the hidden layer. This method was found to be better in terms of PSNR compared to conventional JPEG approach. Durai and Saro [12] have suggested another compression technique using backpropagation method with a cumulative distributed function (CDF). This approach is based on mapping the pixels by estimating the CDF values. However, the decompressed image is fuzzy which is not suggested in medical applications.

To improve the retrieval accuracy, Wan and Kabuka [13] had proposed a neural network approach based on preservation of edges. In this network, quantization levels are used to represent the compressed patterns. The average mean square value is calculated to achieve the compression ratio. In [14], a lossless medical compression technique based on neural network with improved backpropagation method is proposed. From the analysis, it is found that the system exhibits significant performance in compression with low PSNR. Khashman and Dimililer [15] have proposed a medical compression using a neural network with a Haar wavelet compression with nine compression ratios and a supervised neural network that learns to associate the image intensity (pixel values) with a single optimized compression ratio. The limitation of this method is that the image quality is not good which is not tolerable in medical processing applications. To improve the image quality, in [16] neural network with multiresolution method is suggested. This method uses a filter bank that can synthesize the signal accurately from only the reference coefficients that will be well suited for low bitrate coding where the detailed coefficients are coarsely quantized. This approach shows advantages over the conventional approaches for compression at low bitrates, although its performance suffers at high bitrates. For achieving higher bitrates, Mi and Huang [17] presented neural network concepts with principal component analysis. Convergence speed is high for this technique but the image quality is poor. A similar technique is proposed in [18]. The technique includes steps to break down large images into smaller windows and to eliminate redundant information. From the analysis this technique results in achieving a higher compression ratio with the cost of high complexity.

Cottrell et al. [19] developed a multilayered perceptron neural network with backpropagation as the error learning function. This technique results in an optimal compression ratio. Khashman and Dimililer [20] have presented neural network for image compression by DCT transform. Here compression is achieved by DCT coefficients and a supervised neural network that learns to associate the grey image intensity (pixel values) with a single optimized compression ratio. More recently, different image compression techniques were combined with neural network classifier for various applications [21–23]. However, none of these works has achieved optimum compression ratio. To get higher compression ratio, neural network with bipolar coding [24] was proposed. The bipolar coding technique using feed-forward backpropagation neural network converts decimal values into its equivalent binary code and reconverts in decompression phase. Besides higher compression ratio it also preserves the quality of the image. In [25] a similar image compression technique for neural network with GA was suggested. This method mainly focuses on the GA algorithm which uses XOR classification and mapping of small data for compression.

Gaidhane et al. [26] have suggested a neural network based image compression technique with an MLP algorithm for better faster transmission. With this technique some of the information below the threshold value is removed or replaced with zero and therefore more information was removed from the feature vector matrix and hence from image data which results in poor image quality. A similar concept was suggested in [27] which is called vector quantization in which a set of code vectors is generated using the self-organizing feature map algorithm. Then, the set of blocks associated with each code vector is modeled by a cubic surface for better perceptual fidelity of the reconstructed images. AL-Allaf [28] also suggested a similar method, neural network image compression technique. The performance of the suggested method in terms of PSNR, convergence speed, and compression ratio is satisfactory.

For achieving better results, [29] suggests a novel technique, that is, neural network with bipolar interpolation, to balance the tradeoff of speed and quality. With this technique, compression is achieved by selecting primitive and nonprimitive regions to interpolate them. This method was found to be superior to conventional methods in some aspects, such as the clarity and the smoothness in the edge regions as well as the visual quality of the interpolated images. Hui and Yongxue [30] presented a similar neural network concept with Haar wavelet and reconstructed the medical image by wavelet packet. It is based on the fact that wavelet packet domain of the same orientation is often similar and is thus coded by similar code words with a vector quantization algorithm. A neural network approach with arithmetic coding using perceptron neural network to compress the pixel into single value is explained in [31]. A counter propagation neural network has been used to successfully compress and decompress image data. The network also shows robustness for various classes of images.

Mishra and Zaheeruddin [32] have suggested a new fuzzy neural network for medical image compression. This process is based on approximation problem in which it involves determining or learning the input-output relations using numeric input-output data for image compression application. A similar concept was proposed in [33] in which neural network is designed with the modified preprocessing algorithm. The method was divided into two phases. The first part presents the BS-CROI method of image selection and backpropagation image compression in which it is different from traditional ROI. It is found from the analysis that the reconstructed image by this method was promising in terms of PSNR and MSE.

This concept is extended in [34] for better retrieval of image. In this work, neural networks are designed for a combination of cascaded networks with one node in the hidden layer. A redistribution of the gray levels in the training phase is implemented in a random fashion to make the minimization of the mean square error applicable to a broad range of images. From the results of [34] it is found that the performance of cascaded neural networks compared to that of fixed architecture training paradigms is superior especially at high compression ratios.

With the existing approaches for compression, the application for image compression based on advanced intelligence approaches using neural network is observed to be an effective approach for compression. The approach for medical image compression using neural network is developed in the proposed work. In this research the effectiveness of the neural network approach for biomedical image compression is focused.

## 2. Methodology

For medical image compression, in this work an ANN based image compression architecture is developed. In ANN based compression system the image is coded with respect to its pixel values and pixel coordinate. In [35] an approach for medical image compression based on BPNN is proposed. The approach is developed as improved BPNN and is compared with conventional JPEG based coding system. In such an approach an image is first read into a matrix of dimension *m* × *n* and the cosimilar pixel coefficients are searched forming a pair of pixel values of its counts. This approach is similar to the approach of run length coding for the obtained cosimilar pairs; a NN process is carried out, wherein these pairs are given as input to the NN system. The process of NN processing for image compression is briefed as follows.

### 2.1. Image Compression Process

**Step 1 .**

Input image is converted to the matrix format (*I*) containing *X*_*m*,*n*_, where *m* is row and *n* is the column.

**Step 2 .**

Using (*I*), pixel values and the number of occurrences of the neighbouring pixel values are counted and represented by pair values (*P*) as follows:

(1) $$\begin{array}{c} P=\left(U_{1},V_{1}\right)\left(U_{2},V_{2}\right)\left(U_{3},V_{3}\right),\ldots ,\left(U_{i},V_{j}\right), \end{array}$$

where *U* represents pixel values and *V* represents the number of occurrences of the neighboring pixel values.

**Step 3 .**

The pair values (*P*) obtained from the above step can be represented in sequence order (*S*):

(2) $$\begin{array}{c} S=U_{1},V_{1},U_{2},V_{2},U_{3},V_{3},\ldots ,U_{i},V_{j}. \end{array}$$

**Step 4 .**

The sequence order (*S*) can be provided as an input (*X*_*i*_) to the multilayer feed-forward backpropagation neural network:

(3) $$\begin{array}{c} X_{i}=X_{1},X_{2},X_{3},\ldots ,X_{n}. \end{array}$$

**Step 5 .**

Calculate the weight (*W*_*ji*_) using the formula

(4) Wji=∑i=1nXiXiT, where  1≤j≤k;Xi  is  the  input  layer.

**Step 6 .**

The hidden layer of the multilayer feed-forward backpropagation neural network is created by using the formula (*H*_*j*_):

(5) Hj=∑i=1nWijXi, where  1≤j≤k;Xi  is  the  input  layer,Hi=H1,H2,H3,…,Hk.

The result of the *H*_*j*_ obtained refers to the compressed file.

### 2.2. Image Decompression Process

**Step 1 .**

Get (*H*_*j*_) of the multilayer feed-forward backpropagation neural network:

(6) $$\begin{array}{c} H_{j}=H_{1},H_{2},H_{3},\ldots ,H_{k}. \end{array}$$

**Step 2 .**

Calculate the weight (*W*_*ij*_) using the formula

(7) Wij=∑j=1kHjHjT, where  1≤i≤n;Hi  is  the  hidden  layer.

**Step 3 .**

The output layer of the multilayer feed-forward backpropagation neural network is created by using the formula (*Y*_*i*_):

(8) Yi=∑j=1kWij′Hj, where  1≤i≤n;Hi  is  the  hidden  layer,Yi=Y1,Y2,Y3,…,Yn.

**Step 4 .**

The output layer (*Y*_*i*_) is represented by sequence order (*S*):

(9) $$\begin{array}{c} S=U_{1},V_{1},U_{2},V_{2},U_{3},V_{3},\ldots ,U_{i},V_{j}. \end{array}$$

**Step 5 .**

The sequence order (*S*) value can be represented in pair values (*P*). Each pair represents the pixel value and the number of occurrences of the neighbouring pixel values:

(10) $$\begin{array}{c} P=\left(U_{1},V_{1}\right)\left(U_{2},V_{2}\right)\left(U_{3},V_{3}\right),\ldots ,\left(U_{i},V_{j}\right), \end{array}$$

where *U* represents pixel values and *V* represents the number of occurrences of the neighbouring pixel values.

**Step 6 .**

All the pair values (*P*) represented in pixel values are converted into matrix format (*I*).

**Step 7 .**

Now the matrix format (*I*) is converted into the image file format.

Due to this conversion the retrieval accuracy is lower. To improve such estimation accuracy the image must be processed in spectral domain, rather than on direct pixel values. In this conventional approach image is directly processed, so the finer details of biomedical image samples are more or less lost. The multiresolution information is not observed in the previous approach of JPEG2000. So there is a need for a coding technique which presents a high resolution coding resulting in higher estimation accuracy than the JPEG system. The approach of such spectral coding is adopted for medical image compression into NN based coding. In this approach the medical image is first processed to extract the spectral coefficient over which NN is applied and this novel technique of coding results in higher efficiency when compared with existing approaches.

The proposed approach is as outlined below.

In the image compression process, the input image is not processed directly; instead the input image is processed after converting to matrix format and is decomposed to four multiresolution components: *C*_1_ (horizontal coefficients), *C*_2_ (vertical coefficients), *C*_3_ (diagonal coefficients), and *C*_4_ (approximate coefficients).

**Step 8 .**

Input image is converted to the matrix format (*I*), where *I* = *f*(*X*_*m*,*n*_), where *m* is row and *n* is the column.

**Step 9 .**

Using (*I*), decompose the image (*I*) into multiresolution components *C*_1_, *C*_2_, *C*_3_, and *C*_4_ by pyramidal decomposition using discrete wavelet transformation, where *C*_1_ is horizontal coefficients, *C*_2_ is vertical coefficients, *C*_3_ is diagonal coefficients, and *C*_4_ is approximate coefficients.

Further, the steps from 2 to 6 of image compression process and steps from 2 to 7 of decompression processes mentioned in the conventional approach are repeated over the coefficient *C*_*i*_, *i* = 1,2, 3,4.

### 2.3. The Proposed System Architecture

**Figure 1:**
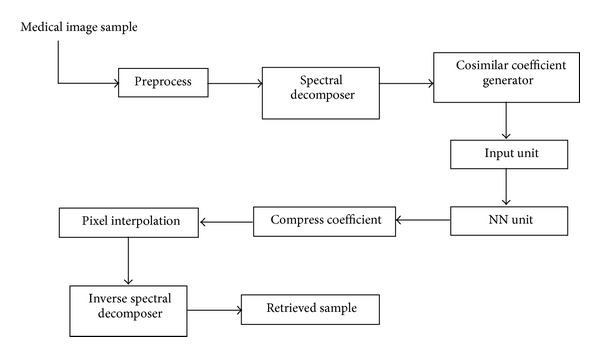
The proposed block diagram.

The functional description of the proposed block diagram (Figure 1) is as follows.

*Preprocess Unit*. This unit reads the medical sample and extracts the gray pixel intensity for processing. The read samples are passed as a pixel array as output of this block and passed for decomposition in spectral decomposer unit.

*Spectral Decomposer Unit*. This unit reads the gray coefficients and performs a pyramidal decomposition to extract the spectral resolutions for a given input sample. The decomposition structured is a 2-dimensional recursive filter bank unit, performing DWT operation. The recursive operation is carried out by the recursive filtration using pairs of successive high and low pass filters.

*Cosimilar Coefficient Generator Unit*. For the obtained coefficient after spectral decomposition, the coefficients which reflect similar spectral coefficients are segregated; these coefficients are called redundant pixel in the image. The suppression of cosimilar coefficient results in first level compression based on redundant information. For the obtained cosimilar coefficients a neural network modelling is developed.

*Input Unit*. This unit reads the selected coefficient and normalizes the coefficients to pass to the neural network. The unit extracts the coefficient in a column-wise manner and is normalized to the maximum pixel value.

*NN Unit*. This unit realizes a feed-forward neural network using the command “newff” in Matlab tool. The NN unit extracts the min-max value of given input and creates a feed-forward neural network taking the least mean learning algorithm. A tangential sigmoid driving function is used as a kernel function for creating this network. The network is created for converging to the error with a goal of 0.1 and with number of epochs = 50. The created network is trained with these coefficient values based on the given input and the created feed-forward network.

*Compress Coefficient*. The coded coefficient after the neural network process is stored into a buffer called compressed coefficient. This formulates an array logic wherein the coded output of the NN is stored for future use.

*Pixel Interpolation*. The compressed data is processed back in this unit, wherein the simulated result of the created neural network is normalized back to its original scale based on the obtained simulated output of the neural network. The retrieved pixel coefficients are rearranged depending on the sequence order as obtained from the encoding side.

*Inverse Spectral Decomposer*. The coefficients obtained from the above units are processed back, where the coefficients are passed back as resolution information to successive high and low pass filter. The recursive output of each level of filtration is added to the other level filtration result and is recursively filtered to obtain a final retrieved level. An inverse DWT approach is followed in this unit.

## 3. Results and Discussion

**Figure 2:**
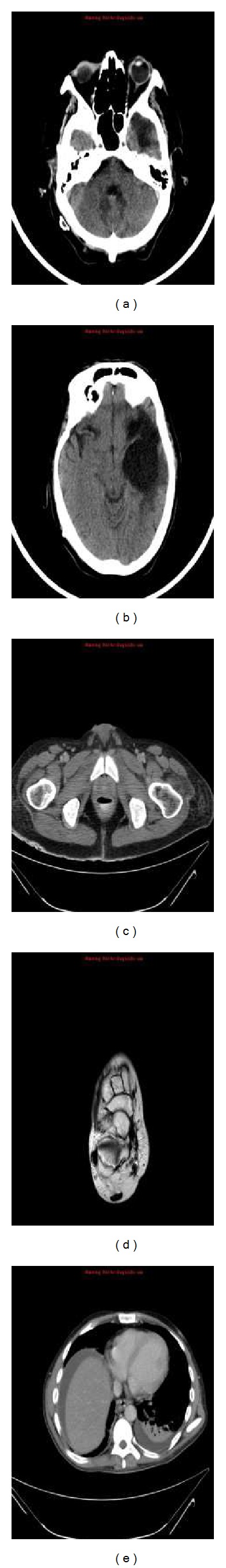
Original image samples Q1, Q2, Q3, Q4, and Q5. Simulation results: image type: medical image; file type: TIFF; test sample: Q1; original size: 87.1 kb; resolution: 512 × 512; compressed size: 40.3 kb; retrieved size: 87.1 kb.

**Figure 3:**
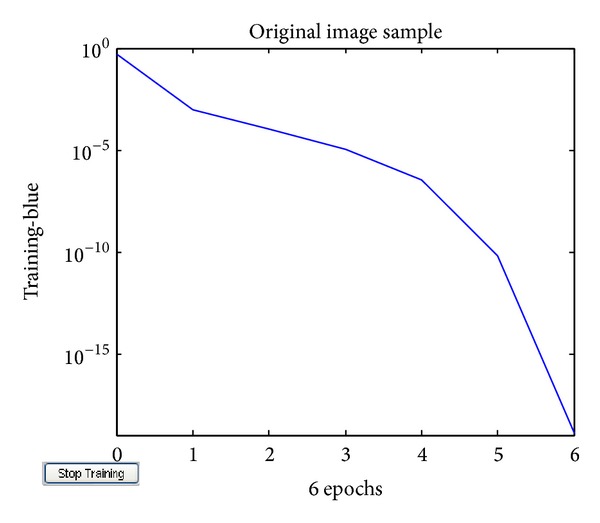
Training error plot for neural system developed.

**Figure 4:**
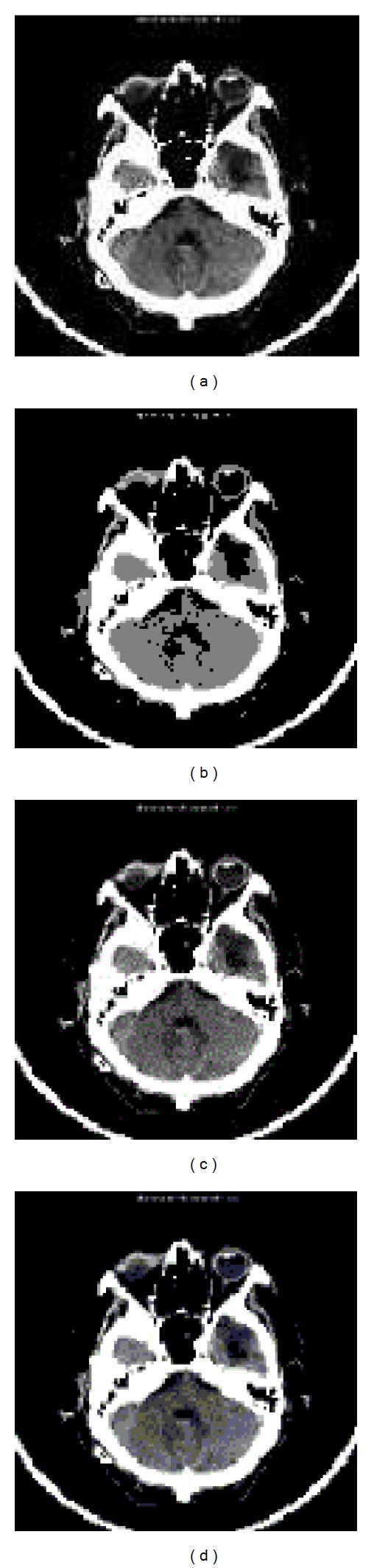
(a) Original processing sample Q1; (b) retrieved image using JPEG2000; (c) retrieved image using improved BPNN; (d) retrieved image using the proposed spectral-BPNN.

**Figure 5:**
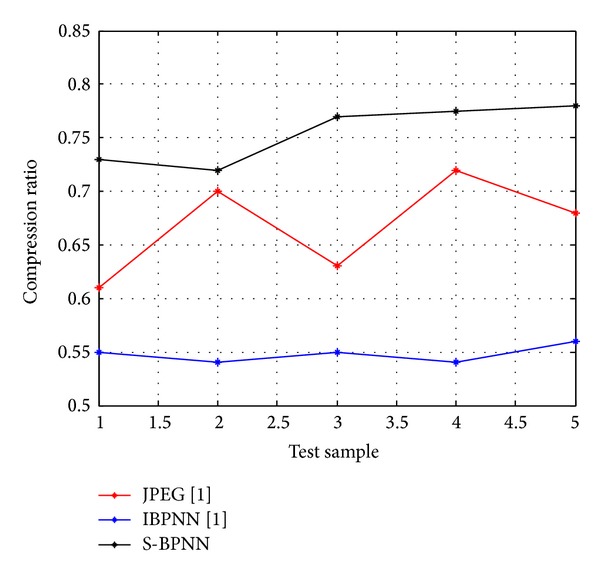
Comparison of compression ratio when the three methods are applied.

**Figure 6:**
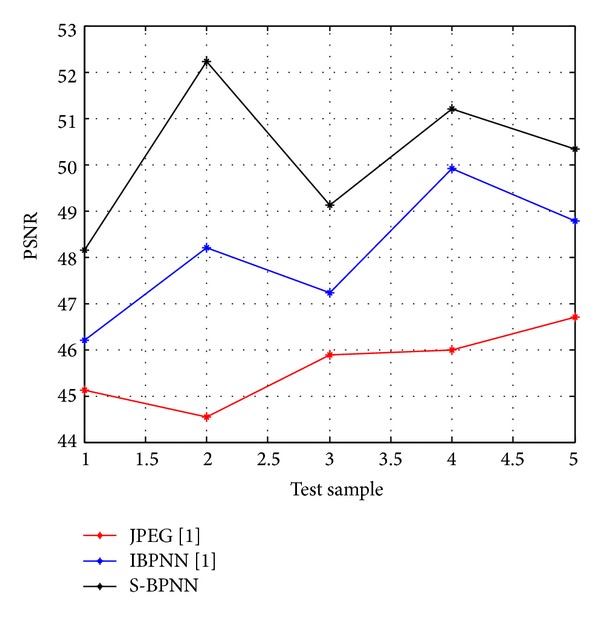
Comparison of PSNR in dB when the three methods are applied.

**Figure 7:**
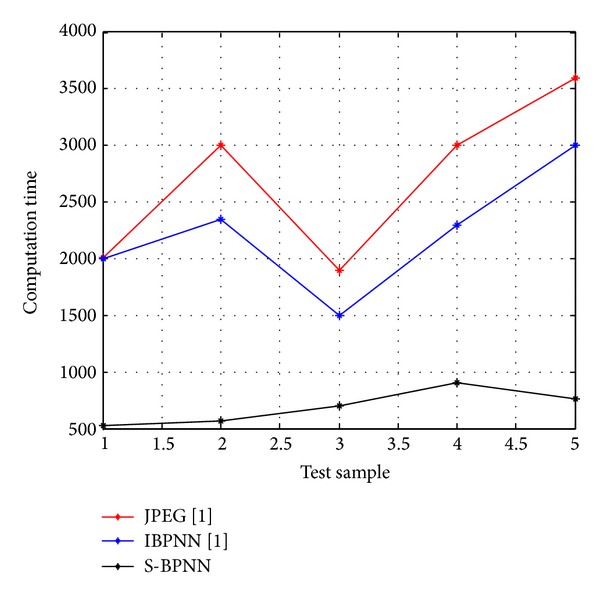
Comparison of computation time in milliseconds when the three methods are applied.

For the evaluation of the suggested approach a simulation model is developed using Matlab and has been tested on various original gray-scale samples of medical images (Figure 2) such as human nerve cells and human body organs of different dimensions collected from hospitals in King Fahd Medical City, Riyadh, Saudi Arabia, with 500 dpi resolution. The training error plot for neural system developed is as shown in Figure 3. The Q1 processing sample was read with various specifications (Figure 4(a)). The output image using conventional JPEG2000 approach is as shown in Figure 4(b). The output image using improved BPNN is as shown in Figure 4(c) and the output image after applying the proposed approach is as shown in Figure 4(d). In [36] the authors compare the proposed approach with the existing approaches with respect to compression ratio and other factors were left for future work. The observations are as shown in Figures 5, 6, and 7.

Comparison plots between five biomedical image samples Q1, Q2, Q3, Q4, and Q5 on *x*-axis with respect to their observed values of compression ratio, PSNR, and computation time for JPEG2000 and improved BPNN and proposed spectral-BPNN approach are illustrated below. The results show that the proposed spectral-BPNN is more efficient than JPEG2000 and improved BPNN for achieving high compression ratio, high PSNR and takes less computation time for all samples of biomedical images.

## 4. Conclusion

This research work implements an enhanced image coding system for biomedical image compression compared to the existing JPEG2000 and other coding techniques. It is observed that the proposed approach is able to achieve good quality performance with a relatively simple algorithm. Since ANN also has the desirable properties resulting from its successive approximation quantization, different topologies were applied to solve the problem. The results obtained from hybrid neural networks found much better results when compared to conventional approaches.

Since this work mainly focuses on gray-scale images, in the future, it can be extended to color medical images by considering regional information such as texture and boundary information, and the observed results can be compared with other standard compression schemes which are used for compression in biomedical imaging.
